# Editorial for the Special Issue on 3D Printing of MEMS Technology

**DOI:** 10.3390/mi14122195

**Published:** 2023-11-30

**Authors:** Andrea Ehrmann

**Affiliations:** Faculty of Engineering Sciences and Mathematics, Bielefeld University of Applied Sciences and Arts, 33619 Bielefeld, Germany; andrea.ehrmann@hsbi.de

Microelectromechanical systems (MEMS) combine electrical and mechanical functions and are nowadays broadly applied in many technology fields, often as sensors or actors [1]. Opposite to microelectronics and micromechanics, standardization of the production processes is still relatively low. While this poses challenges for the development of new MEMS for specific applications, it also calls for the use of additive manufacturing with its high degree of freedom in design and the large chance of individualization to produce new MEMS [2].

Additive manufacturing, or 3D printing, belongs to the emerging technologies of our time. While previously they were mostly used for rapid prototyping, the technology has been in rapid production for a long time, especially for complicated objects or those with small lot sizes [3]. Most recently, new 3D printing technologies enable the printing of the smallest features on micro- or even nano-scales [4]. At the same time, well-known problems like the waviness of fused deposition modeling (FDM) printed parts, the missing long-term stability of some typical printing materials, or the reduced mechanical properties of 3D-printed objects still exist and have to be investigated in detail to enable the optimization of these parameters [5].

This Special Issue focusses on all topics dealing with the 3D printing of microelectromechanical systems (MEMS), such as new or advanced features enabled by 3D printing compared to conventional technologies, but also the challenges which still exist of using 3D printing technologies for MEMS and new approaches for how to overcome them.

One of the challenges addressed by the papers in this Special Issue is dimensional accuracy and surface roughness, e.g., in PolyJet printing (Contribution 1) or powder bed fusion (Contribution 2). A high mixing efficiency (Contribution 3) was aimed to be created, as well as the creation of specific optical properties (Contribution 4). Amongst the investigated materials, polyvinyl alcohol (PVA) was investigated as a possible sacrificial material for MEMS devices (Contribution 5), as well as photo-sintered magnetic strontium ferrite samples (Contribution 6), polymethylmethacrylate (PMMA) microfluidic chips (Contribution 7), as well as the in situ annealing of semiconducting ZnO thin films (Contribution 8). Specific structures (Contribution 9) are investigated as well as sensors (Contribution 10), actuators (Contribution 11), and electronic devices (Contribution 12), partly combining an experiment and simulation (Contribution 13). Additionally, a review of the recent developments and applications of 3D-printed MEMSs technology is given (Contribution 14).

To conclude, the papers collected in this Special Issue provide an overview of different additive manufacturing methods, such as fused deposition modeling, photolithography, PolyJet, aerosol jet or inkjet, and materials including diverse polymers and metals. They describe the development of microfluidic and colorimetric devices, micro-resonators and micro-beams, sensors and actuators, and resistors and micro-heaters, as well as the corresponding challenges and proposed solutions. We hope that these papers will inspire more research in the highly topical research area of 3D printing MEMS.

## References

[1] Tanaka M. (2007). An industrial and applied review of new MEMS devices features. Microelectron. Eng..

[2] Blachowicz T., Ehrmann A. (2020). 3D printed MEMS Technology—Recent Developments and Applications. Micromachines.

[3] Ben-Ner A., Siemsen E. (2017). Decentralization and localization of production: The organizational and economic consequences of additive manufacturing (3D printing). Calif. Manag. Rev..

[4] Ertugrul I. (2020). The fabrication of micro beam from photopolymer by digital light processing 3D printing technology. Micromachines.

[5] Kozior T., Bochnia J. (2020). The influence of printing orientation on surface texture parameters in powder bed fusion technology with 316L steel. Micromachines.

